# Association of glycated hemoglobin with non-alcoholic fatty liver disease patients and the severity of liver steatosis and fibrosis measured by transient elastography in adults without diabetes

**DOI:** 10.1186/s12902-022-01134-z

**Published:** 2022-08-31

**Authors:** Yilian Xie, Weiliang Kong, Xuepeng Wang, Zhouxiao Wu

**Affiliations:** 1 Department of Infectious Diseases, Ningbo First Hospital, Ningbo, 315010 Zhejiang China; 2 Department of Hepatology, Ningbo First Hospital, Ningbo, 315010 Zhejiang China; 3 Department of Respiratory and Critical Care Medicine, Ningbo First Hospital, Ningbo, 315010 Zhejiang China; 4 School of Medicine, Ningbo University, Ningbo, China

**Keywords:** HbA1c, NAFLD, NHANES, Steatosis, Transient elastography

## Abstract

**Background:**

Type 2 diabetes mellitus (T2DM) is a well-known independent risk factor for non-alcoholic fatty liver disease (NAFLD). However, research exploring the association between blood glucose management and the risk of NAFLD status in subjects without diabetes was insufficient. This study aimed to explore the association of glycated hemoglobin (HbA1c) with NAFLD status and the severity of liver steatosis and fibrosis in non-diabetic people.

**Methods:**

A cross-sectional analysis was conducted on 2998 non-diabetic American adults using data from the National Health and Nutrition Examination Survey (NHANES) 2017–2018 cycle. We used multivariable logistic regression models to evaluate the association between HbA1c and NAFLD status and the severity of liver steatosis and fibrosis. Interaction and stratified analyses were additionally performed.

**Results:**

The multivariate regression analyses showed that HbA1c was associated independently with NAFLD status in all the models (model1: OR = 2.834, 95%CI: 2.321, 3.461; model 2: OR = 2.900, 95%CI: 2.312, 3.637 and model 3: OR = 1.664, 95%CI: 1.284, 2.156). We further performed the interaction and stratified analyses and discovered a significant interaction between HbA1c and BMI (P_*interaction*_ < 0.05). Finally, a robust link was shown between HbA1c level and the severity of liver steatosis, which was mainly significant in the prediabetes group, while the correlation was not significant in HbA1c level and severity of liver fibrosis after controlling for all the potential confounders.

**Conclusions:**

We concluded that HbA1c level was positively correlated to the risk of developing NAFLD in a large non-diabetic American population. Moreover, HbA1c level was associated with the severity of liver steatosis in subjects with prediabetes, suggesting that routine screening for HbA1c among individuals with prediabetes is necessary.

## Background

In developed nations, non-alcoholic fatty liver disease (NAFLD) has become one of the most prevalent causes of chronic liver disease, with a global prevalence of approximately 25–30% [1]. In North America, the prevalence of NAFLD has even reached 35.3% [2] and there will be 100.9 million patients in 2030 [3]. NAFLD consists of a continuous process of liver tissue lesions, from steatosis to non-alcoholic steatohepatitis (NASH), which is characterized by hepatocyte ballooning, lobular inflammation, and/or fibrosis, and eventually cirrhosis or even hepatocellular carcinoma (HCC) [4].

Numerous studies have demonstrated a complex relationship between NAFLD and metabolic diseases. It is believed that metabolic diseases, especially type 2 diabetes mellitus (T2DM) can promote NAFLD, and conversely, NAFLD can increase the incidence of type 2 diabetes [5, 6]. Stefano Ciardullo et al. reported that the prevalence of NAFLD was 33.7% in the general population but 74.9% in subjects with diabetes based on data from the National Health and Nutritional Examination Survey (NHANES) 2017–2018 cycle [7, 8]. Meanwhile, in an updated meta-analysis that included 80 studies from 20 different nations, the prevalence of NAFLD among patients with T2DM worldwide was 55.5%. However, the publications between blood glucose control and NAFLD risk in non-diabetic individuals were not enough. Glycosylated hemoglobin (HbA1c) is a typical glycosylated protein and is widely used in the assessment of glycemic control [9]. It is produced through the interaction of hemoglobin and blood glucose and can be used to reflect the average blood glucose level over 2–3 months [10]. Several studies in Asia have demonstrated that HbA1c was positively associated with NAFLD status among participants without diabetes [11–14], but little is known about western populations and whether a special population exists among different subgroups. Moreover, comprehensive data about the association between HbA1c and the severity of liver steatosis and fibrosis in NAFLD individuals without diabetes is limited.

Although liver biopsy is still the gold standard for diagnosing and assessing the severity of liver steatosis and NASH, other non-invasive and cost-effective procedures have been extensively researched and reported [15]. Transient elastography (TE) is regarded as a promising and regularly used non-invasive approach for liver steatosis and fibrosis quantification [16] and has been utilized to identify liver steatosis and fibrosis in the general population [17, 18]. The controlled attenuation parameter (CAP) value, in particular, rises with the degree of liver steatosis and can be applied to detect steatosis of more than 5% [19, 20]. Simultaneously, a higher liver stiffness measurement (LSM) value implicates more severe fibrosis [19].

Herein, we explored the association between HbA1c and NAFLD in the adult American population without diabetes using data from the latest cycle of NHANES. And we additionally evaluated the relationships between HbA1c and the severity of liver steatosis and fibrosis measured by TE.

## Methods

### Study population

Our current investigation is based on information obtained from the NHANES 2017–2018 cycle. NHANES is a broadly well-designed cross-sectional survey of non-institutionalized citizens in the United States [21]. A complicated, stratified, multistage probabilistic cluster sampling method was applied to acquire samples representing the whole nation for each cycle [21]. The investigation protocol for NHANES was granted ethical approval by the National Center for Health Statistics (NCHS) Research Ethics Review Board and written consent was signed by all members.

### Study design

**Fig. 1:**
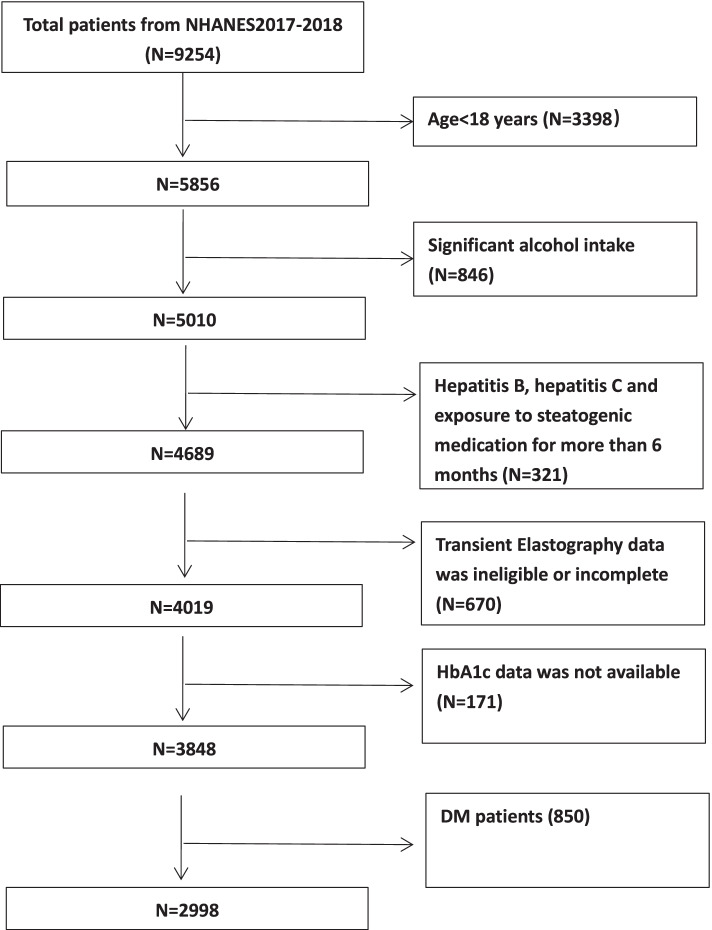
Flow-chart of the study samples

There was a total of 9254 participants in the NHANES 2017–2018 cycle. The exclusion criteria of our study were mainly based on the definition of NAFLD [22]. We excluded 3398 participants under the age of 18 and 846 participants who had significant alcohol intake (male > 21 drinks per week and female > 14 drinks per week) [22]. Then we excluded 321 participants with viral hepatitis B (hepatitis B surface antigen-positive) and hepatitis C ((hepatitis C antibody positive), and those who had taken steatogenic medication for more than half a year. Steatogenic medication involves tamoxifen, amiodarone, methotrexate, valproate, antiretroviral drugs, and corticosteroid [22]. In addition to these exclusion criteria, we also exclude 1087 participants without available HbA1c data and TE data (ineligible or incomplete). Meanwhile, 850 patients with diabetes were ruled out. Diabetes is diagnosed as self-reported diabetes (diagnosed by a physician or other health professional), oral hypoglycemic drugs or insulin usage, fasting glucose ≥ 126 mg/dl, or HbA1c level ≥ 6.5% [7, 23, 24]. Ultimately a total of 2998 people were involved in our survey (Fig. 1).

### Definition of exposure and outcome variables

HbA1c was the exposure variable in our study. It was tested by the Tosoh Automated Glycohemoglobin Analyzer HLC-723G8 using specialized software. CAP and LSM values were two continuous outcome variables measured by Liver ultrasound TE while NAFLD was a categorical outcome variable defined as CAP values ≥ 263 dB/m [25]. NHANES health technicians performed the TE exam after being trained and authorized by the equipment manufacturer and NHANES staff. The tests were carried out following the manufacturer's instructions. Liver ultrasound TE exams were regarded as valid if at least 10 LSM values were obtained after fasting for at least 3 h, with an interquartile (IQR) range/median < 30% [7].

### Definition of covariates

Sex, race (non-Hispanic white, non-Hispanic black, Hispanic, and other race), hypertension, smoking status (current smoker, former smoker, and never smoked divided by smoking self-report), activity level, dyslipidemia, and homeostasis model assessment of insulin resistance (HOMA-IR) were all included as categorical covariates in our analysis. Age, body mass index (BMI), waist circumference (WC), and hemoglobin were the continuous covariates in our study.

Hypertension was categorized if any of the conditions below existed: (1) systolic blood pressure ≥ 140 mmHg or diastolic blood pressure ≥ 90 mm Hg. (2) Anti-hypertensive medicine is currently being used. (3) Hypertension that is self-reported. Overweight was defined as BMI values of 25–29.9 kg/m^2^, whereas obesity was defined as BMI values ≥ 30 kg/m^2^ [26]. The activity level was classified as active, moderate, and inactive levels according to the Global Physical Activity Questionnaire. This approach has been described previously [27]. Dyslipidemia was defined as plasma high density lipoprotein (HDL)-cholesterol < 40 mg/dL for male and < 50 mg/dL for female, plasma triglycerides ≥ 150 mg/dL, or with particular pharmacological therapy [28]. HOMA-IR was divided into normal insulin resistance (HOMA-IR < 2.5) and high insulin resistance (HOMA-IR ≥ 2.5), respectively [29]. Prediabetes was defined as self-reported prediabetes (diagnosed by a physician or other health professional), HbA1c 5.7 to 6.4%, or fasting glucose 100 to 125 mg/dL [30, 31].

### Statistical analysis

NHANES sample weights were taken into consideration as recommended by NCHS. Categorical variables were presented as percentages, while continuous variables were expressed as weighted mean ± standard deviation. Multivariate logistic regressions were applied to identify an independent relationship between HbA1c and the odds of NAFLD after adjusting for potential clinical confounders. Multivariate linear regression analysis was utilized to evaluate correlations between HbA1c and the severity of liver steatosis and fibrosis based on liver CAP value and LSM value, respectively. As a sensitivity analysis, we categorized HbA1c into quartiles and calculated *P*-values ​​for trends test. Furthermore, we subdivided the subjects into two groups: normoglycemic levels and prediabetes stage, and performed subgroup analysis. Interaction and stratified analyses were conducted based on sex, age, and BMI. Three models were constructed in our study: model 1: no covariates were adjusted; model 2: sex, age, and race were adjusted; model 3: age, sex, race, hypertension, smoking status, BMI, WC, activity level, hemoglobin, dyslipidemia, and HOMA-IR were adjusted. The software R (http://www.R-project.org) and EmpowerStats (http://www.empowerstats. com) were performed for all analyses, with a *P*-value < 0.05 considered statistically significant.

## Results

Baseline characteristics of 2998 adult participants without diabetes based on the status of NAFLD were presented in Table 1. Participants with NAFLD were elder, more likely to be men, inactive and ever smoker, more non-Hispanic White or Hispanic, had more severe liver steatosis and fibrosis, higher BMI and WC, higher total cholesterol, triglyceride, fasting glucose, HbA1c, alanine aminotransferase (ALT), aspartate aminotransferase (AST), alkaline phosphatase (ALP), gamma-glutamyltranspeptidase (GGT), and had lower HDL-cholesterol levels (*p* < 0.05 for each). They showed a higher prevalence of hypertension. Non-Hispanic Black was lower in liver steatosis participants. Nonetheless, no significant differences were observed in serum creatinine levels.

**Table 1:** Weighted characteristics of the NAFLD and non-NAFLD groups

	Non-NAFLD (*n* = 1679)	NAFLD (*n* = 1319)	*P* value
Age(years)	44.1 ± 18.1	49.8 ± 16.2	< 0.0001
Age (%)			< 0.0001
18-39y	47.20	30.50	
40-59y	28.70	40.20	
60-80y	24.10	29.20	
Sex (%)			< 0.0001
Male	45.00	53.20	
Female	55.00	46.80	
Race (%)			< 0.0001
Non-Hispanic White	62.80	63.80	
Non-Hispanic Black	12.90	9.20	
Hispanic	5.60	10.50	
Other Race	18.70	16.50	
Smoking behavior (%)			0.0116
Current smoke	14.70	11.80	
Ever smoke	21.80	25.40	
Never smoke	63.50	62.80	
Hypertension (%)			< 0.0001
No	73.80	52.20	
Yes	26.20	47.80	
activity level			< 0.0001
inactive	30.90	39.70	
Moderate	10.90	8.90	
active	58.20	51.40	
BMI (Kg/m^2^)	26.1 ± 5.4	32.6 ± 6.9	< 0.0001
BMI (%)			< 0.0001
BMI < 25	45.70	7.80	
25 ≤ BMI < 30	35.30	31.80	
BMI ≥ 30	19.00	60.40	
WC (cm)	91.1 ± 13.5	107.6 ± 14.5	< 0.0001
ALT (IU/L)	19.4 ± 14.4	25.8 ± 16.6	< 0.0001
AST (IU/L)	21.0 ± 10.8	22.4 ± 11.2	0.0003
ALP(IU/L)	74.1 ± 24.2	79.0 ± 21.9	< 0.0001
GGT (IU/L)	21.8 ± 21.5	33.3 ± 38.9	< 0.0001
Serum creatinine (mg/dL)	0.9 ± 0.2	0.9 ± 0.4	0.1881
Fasting glucose(mg/dL)	90.1 ± 8.2	94.6 ± 9.3	< 0.0001
HbA1c (%)			< 0.0001
Q1(4.1–5.1%)	27.6	16.1	
Q2(5.2–5.4%)	34.2	28.5	
Q3(5.5–5.6%)	19.7	20.9	
Q4(5.7–6.4%)	18.5	34.5	
Total cholesterol (mg/dL)	186.0 ± 38.2	195.4 ± 38.2	< 0.0001
Triglyceride (mg/dL)	109.8 ± 61.3	161.8 ± 118.1	< 0.0001
HDL-cholesterol (mg/ dL)	57.1 ± 14.1	50.1 ± 13.5	< 0.0001
CAP (dB/m)	214.2 ± 33.7	310.0 ± 35.6	< 0.0001
LSM (kPa)	4.9 ± 3.3	6.1 ± 5.2	< 0.0001

Mean ± SD was for continuous variables. The *p*-Value was calculated by weighted linear regression model. % was for categorical variables. The *p*-Value was calculated by the weighted chi-square test

*Abbreviations*: *NAFLD* Non-alcoholic fatty liver disease, *BMI* Body mass index, *WC* Waist circumference, *ALT* Alanine aminotransferase, *AST* Aspartate aminotransferase, *ALP* Alkaline phosphatase, *GGT* Gamma-glutamyl transpeptidase, *HbA1c* Glycosylated hemoglobin A1c, *HDL* High-density lipoprotein, *LSM* Liver stiffness measurement, *CAP* Controlled attenuation parameter

Table 2 presented the results of the multivariate regression analysis between HbA1c and the prevalence of NAFLD. In model 1, HbA1c was positively correlated to NAFLD status (OR = 2.834, 95%CI: 2.321, 3.461). This positive association was persistent in model 2 (OR = 2.900, 95%CI: 2.312, 3.637) and model 3 (OR = 1.664, 95%CI: 1.284, 2.156) after adjusting for potential confounders. When HbA1c was converted from a continuous variable to a categorical variable (quartiles), participants in quartile2 (HbA1c 5.2–5.4%), quartile3 (HbA1c 5.5–5.6%) and quartile4 (HbA1c 5.7–6.4%) were associated with 13.6%, 9.9%, 62% higher odds of being NAFLD, respectively, compared with quartile1 (HbA1c 4.1–5.1%). A significant linear trend was observed for the correlation between quartiles of HbA1c and NAFLD status (*P* < 0.001). These results suggested that people without diabetes were more likely to develop NAFLD with higher HbA1c than those with lower HbA1c.

**Table 2:** Multivariable odds ratio (OR) for NAFLD status based on HbA1c

	Model 1 OR (95% CI), *P* value	Model 2 OR (95% CI), *P* value	Model 3 OR (95% CI), *P* value
HbA1c	2.834 (2.321, 3.461) < 0.001	2.900 (2.312, 3.637) < 0.001	1.664 (1.284, 2.156) < 0.001
Q1(4.1–5.1%)	Reference	Reference	Reference
Q2(5.2–5.4%)	1.225 (0.980, 1.532) 0.075	1.186 (0.944, 1.491) 0.143	1.136 (0.870, 1.484) 0.348
Q3(5.5–5.6%)	1.474 (1.161, 1.870) 0.001	1.403 (1.092, 1.804) 0.008	1.099 (0.820, 1.473) 0.528
Q4(5.7–6.4%)	2.606 (2.101, 3.234) < 0.001	2.596 (2.043, 3.299) < 0.001	1.620 (1.225, 2.142) < 0.001
P for trend	< 0.001	< 0.001	< 0.001

Model 1: no covariates were adjusted

Model 2: sex, age, and race were adjusted

Model 3: sex, age, race, hypertension, smoking status, BMI, WC, activity level, hemoglobin, dyslipidemia, and HOMA-IR were adjusted

*Abbreviations*: *NAFLD* Non-alcoholic fatty liver disease, *HbA1c* Glycosylated hemoglobin, *BMI* Body mass index, *WC* Waist circumference, *HOMA-IR* Homeostasis model assessment of insulin resistance

**Fig. 2:**
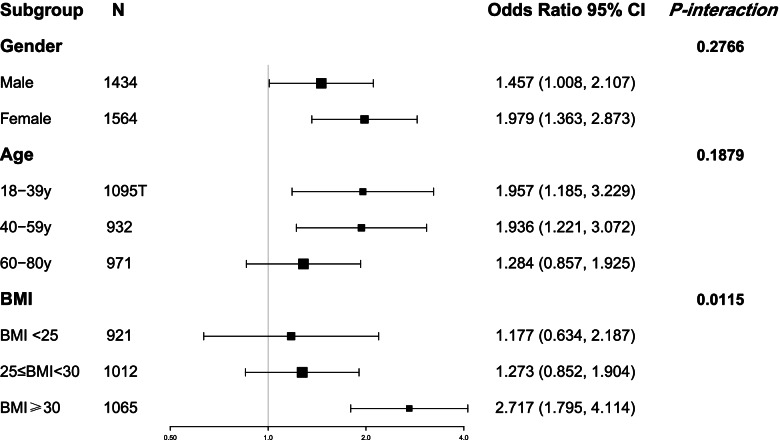
Multivariable odds ratio (OR) for NAFLD status based on HbA1c stratified by sex, age and BMI. Each stratification adjusted for all the factors (sex, age, race, hypertension, smoking status, BMI, WC, activity level, hemoglobin, dyslipidemia, and HOMA-IR) except the stratification factor itself. Abbreviations: NAFLD, Non-alcoholic fatty liver disease; HbA1c, glycosylated hemoglobin; BMI, body mass index; WC, waist circumference; HOMA-IR, homeostasis model assessment of insulin resistance

To further demonstrate the deep relationship between HbA1c and NAFLD status, interaction tests were conducted in the designated subgroups (Fig. 2). Among these subgroups, we discovered a significant interaction between HbA1c and BMI (*P*_interaction_ < 0.05). The risk association was significantly higher in obese individuals (BMI ≥ 30 kg/m^2^). Moreover, the interaction tests for age and sex were not significant (*P*_interaction_ > 0.05).

Table 3 showed the associations between HbA1c and severity of liver steatosis based on CAP value. HbA1c was significantly positively correlated with the severity of liver steatosis, and this association remained unchanged in model 1 (β = 37.448, 95%CI: 31.771, 43.125), model 2 (β = 34.472, 95%CI: 28.301, 40.643) and model 3 (β = 13.437, 95%CI: 8.282, 18.592), with a P for trend of < 0.001 (Table 3). We further subdivided the subjects into two groups: normoglycemic levels and prediabetes stage. The association remained positive in the prediabetes group (β = 18.905, 95%CI: 10.737, 27.074), but did not remain significant in the normoglycemic levels group (β = -2.230, 95%CI: -10.853, 6.393), *P*_interaction_ < 0.001.

**Table 3:** Associations between HbA1c and liver steatosis based on CAP value

	Model 1 *β* (95% CI), *P* value	Model 2 *β* (95% CI), *P* value	Model 3 *β* (95% CI), *P* value
HbA1c	37.448 (31.771, 43.125) < 0.001	34.472 (28.301, 40.643) < 0.001	13.437 (8.282, 18.592) < 0.001
Q1(4.1–5.1%)	Reference	Reference	Reference
Q2(5.2–5.4%)	4.832 (-0.790, 10.454) 0.092	2.777 (-2.750, 8.304) 0.32483	-1.596 (-6.062, 2.870) 0.484
Q3(5.5–5.6%)	14.606 (8.349, 20.864) < 0.001	12.324 (5.934, 18.714) < 0.001	2.979 (-2.221, 8.179) 0.262
Q4(5.7–6.4%)	35.043 (29.137, 40.950) < 0.001	31.389 (25.068, 37.709) < 0.001	12.209 (6.952, 17.467) < 0.001
P for trend	< 0.001	< 0.001	< 0.001
Subgroup analysis			
Normoglycemic levels (*n* = 1635)	15.517 (5.248, 25.786) 0.003	5.843 (-4.707, 16.392) 0.278	-2.230 (-10.853, 6.393) 0.612
Prediabetes (*n* = 1363)	18.423 (9.287, 27.559) < 0.001	30.390 (20.862, 39.918) < 0.001	18.905 (10.737, 27.074) < 0.001
* P*_interaction_			< 0.001

Model 1: no covariates were adjusted

Model 2: sex, age, and race were adjusted

Model 3: sex, age, race, hypertension, smoking status, BMI, WC, activity level, hemoglobin, dyslipidemia, and HOMA-IR were adjusted

*Abbreviations*: *NAFLD* Non-alcoholic fatty liver disease, *HbA1c* Glycosylated hemoglobin, *BMI* Body mass index, *WC* Waist circumference, *HOMA-IR* Homeostasis model assessment of insulin resistance

The association between HbA1c and severity of liver fibrosis in subjects with NAFLD was positive in model 1 (β = 1.063, 95%CI: 0.293, 1.832) and model 2 (β = 1.470, 95%CI: 0.637, 2.304). However, this association was no longer significant after controlling for all the confounders (β = 0.461, 95%CI: -0.337, 1.258) with a *P for trend* of 0.432 (Table 4).

**Table 4:** Associations between HbA1c and liver stiffness based on LSM value in patients with NAFLD

	Model 1 *β* (95% CI), *P* value	Model 2 *β* (95% CI), *P* value	Model 3 *β* (95% CI), *P* value
HbA1c	1.063 (0.293, 1.832) 0.007	1.470 (0.637, 2.304) < 0.001	0.461 (-0.337, 1.258) 0.258
Q1(4.1–5.1%)	Reference	Reference	Reference
Q2(5.2–5.4%)	0.043 (-0.832, 0.919) 0.922	0.135 (-0.745, 1.015) 0.764	-0.009 (-0.828, 0.811) 0.983
Q3(5.5–5.6%)	0.202 (-0.730, 1.134) 0.671	0.538 (-0.419, 1.495) 0.270	0.106 (-0.794, 1.007) 0.817
Q4(5.7–6.4%)	0.781 (-0.067, 1.628) 0.071	1.090 (0.193, 1.986) 0.017	0.275 (-0.572, 1.121) 0.525
P for trend	0.030	0.005	0.432

Model 1: no covariates were adjusted

Model 2: sex, age, and race were adjusted

Model 3: sex, age, race, hypertension, smoking status, BMI, WC, activity level, hemoglobin, dyslipidemia, and HOMA-IR were adjusted

*Abbreviations*: *NAFLD* Non-alcoholic fatty liver disease, *HbA1c* Glycosylated hemoglobin, *BMI* Body mass index, *WC* Waist circumference, *HOMA-IR* Homeostasis model assessment of insulin resistance

## Discussion

This article mainly explored the associations of HbA1c with NAFLD status and the severity of liver steatosis and fibrosis measured by TE in adults without diabetes in the extensive, nationwide cross-sectional research. Our results revealed that high HbA1c levels were linked to a higher risk of NAFLD status in American adults (OR = 1.664, 95%CI: 1.284, 2.156), and this risk association was more significant in obese individuals (OR = 2.717, 95%CI: 1.795, 4.114; *P*_interaction_ = 0.0115). Another important conclusion of this study was that the severity of liver steatosis aggravated when HbA1c levels increased in NAFLD adults with prediabetes. Despite this, the relationship was not significant between the HbA1c level and the severity of liver fibrosis after adjusting for all the potential covariates. To our knowledge, this is the biggest sample size of research on the association between HbA1c and NAFLD in a non-diabetic American population.

Over the past few decades, NAFLD has been reported to have a deep relationship with components of metabolic syndrome (particularly type 2 diabetes and hypertension) [32, 33]. T2DM was recognized as a risk factor for the development of NAFLD [34]. Nonetheless, whether glycemic levels in people without T2DM are linked to the risk of developing NAFLD has not been commonly discussed. In a large cross-sectional study involving 99,969 non-diabetic Korean adults, the results showed the risk of NAFLD development increased with higher HbA1c levels [11]. Meanwhile, in a longitudinal cohort study comprising 4,273 Chinese adults, increased glycemic levels (fasting and 2-h glucose) within the non-diabetic range were negatively linked with the resolution of NAFLD [13]. Additionally, Chao Yu et al. and Han Ma et al. reported the same positive association between HbA1c and NAFLD in non-diabetic Chinese persons aged 20–65 years old and senior people aged 65 years old and older, respectively [12, 14]. Our findings were consistent with these results. It was noteworthy that NAFLD cases in our study were ascertained through TE, therefore the diagnosis of NAFLD can be more accurate than the hepatic ultrasonography performed in those previous studies.

In addition, we extend the evidence by interaction and stratified analyses according to sex, age, and BMI. The subgroup analysis showed that the risk association between HbA1c and NAFLD in obese adults was considerably higher than that in non-obese individuals. As is well known, NAFLD is widespread in people with metabolic syndrome. Obesity is a key risk factor for NAFLD, and it has a favorable relationship with both the existence of NAFLD and the course of the disease [35]. Obesity and diabetes patients have been recognized to have a high incidence of hepatic steatosis, cryptogenic cirrhosis, and a significant risk of developing HCC [35]. On the other hand, obesity without T2DM also correlates to a higher fat content in body tissues. Increased BMI has been linked to insulin resistance and elevation in HbA1C [36].

Another important result of our study is that we discovered a significant relationship between HbA1c level and severity of liver steatosis in a large American NAFLD population without diabetes. We are constrained in making comparisons to the literature since little is known about how glycemic management affects the severity of liver steatosis and fibrosis and the related risk of NAFLD development. The results of recent case–control research involving 450 people in Pakistan indicated that the severity grades of steatosis, which were graded based on fatty infiltration identified on ultrasonography, are substantially correlated with HbA1c levels [37]. In another longitudinal cohort study including 713 subjects with biopsy-proven NAFLD or NASH, Anastasia-Stefania Alexopoulos et al. demonstrated that increased mean HbA1c was related to a higher grade of steatosis, ballooned hepatocytes, and increased fibrosis stage [38]. Unfortunately, neither of these two studies focused on the non-diabetic population. Chao Yu et al. [12]. conducted a cross-sectional study, which revealed high HbA1c levels to be independently related to an elevated risk of advanced fibrosis in Chinese NAFLD patients without diabetes. They applied NAFLD Fibrosis Score (NFS) to assess the liver fibrosis in their article, whereas the severity of liver steatosis was not evaluated. In our study, we used TE, which was considered the best diagnostic performance for the noninvasive assessment of liver fibrosis in NAFLD patients [39], to evaluate the severity of liver steatosis and fibrosis of NAFLD. We found the degree of liver steatosis developed with the increasing level of HbA1c, which was mainly predominate in the prediabetes group, suggesting that HbA1c level could be a potential biomarker for liver steatosis management of NAFLD patients with impaired glucose regulation. Nonetheless, the relationship between HbA1c level and liver fibrosis was not independently significant in our study. Further studies were continued needed to be done.

Researchers have demonstrated that HbA1c levels and the development of NAFLD had many pathophysiologically important linkages. In addition to the creation of advanced glycation end-products, hyperglycemic episodes can disrupt lipid metabolism and result in increased synthesis of triacylglycerols (TAGs), which tend to deposit in many organs of the body, including the liver. TAG deposition in the liver can cause fatty liver and HbA1C may be causally connected with NAFLD [40]. Hepatic steatosis, in turn, may cause the liver to generate proatherogenic and proinflammatory mediators, aggravating hepatic and systemic insulin resistance [41]. The gene-small molecule interaction networks may also explain the relationship between hyperglycemia and NAFLD [42].

The principal strength of our study is that we enrolled the largest cohort of non-diabetic American participants focusing on the correlation between HbA1c and NAFLD status. Moreover, we performed subgroup and interaction analyses, and is the first study, to our knowledge, reported a significant relationship between HbA1c level and severity of liver steatosis based on the TE. Nevertheless, our study had several limitations. First, due to the nature of the cross-sectional design, we are unable to determine the causal association between HbA1c and NAFLD. However, accumulating evidence supports the link between NAFLD and T2DM is convoluted and the relationship seems to be bidirectional [5, 6]. Second, NAFLD status was defined as CAP values ≥ 263 dB/m through TE, but not liver biopsy, which may result in bias in the inclusion of NAFLD patients. In addition, there is presently no clear cutoff rule for the CAP score. However, liver biopsy is invasive and has bleeding risk while TE is non-invasive and cost-effective. TE was considered the best diagnostic performance for liver steatosis and fibrosis quantification in NAFLD patients [16, 39]. We also selected the most well-established CAP score cutoff value from several well-conducted studies [17, 25]. Third, certain participants, particularly obese and elderly people, who did not complete or just partially completed TE were eliminated from our study. This might lead to selection bias. Fourth, self-reported confounders may be subject to self-report bias. Moreover, the database in NHANES 2017–2018 lacks 2-h glucose or random glucose, which may lead to selection bias for diabetes and prediabetes. Finally, there is still a chance of bias caused by other potential confounding factors that we did not adjust for.

## Conclusions

In conclusion, our study indicated that a high HbA1c level was independently associated with the risk of developing NAFLD in a large non-diabetic American population. HbA1c level was also correlated with the severity of liver steatosis in subjects with prediabetes, suggesting that HbA1c could be a possible indicator for the management of NAFLD patients with prediabetes.

## Data Availability

The datasets collected and analyzed during the current study are available on the NHANES website (http://www.cdc.gov/nchs/nhanes.htm).

## Abbreviations

T2DM: Type 2 diabetes mellitus
NAFLD: Non-alcoholic fatty liver disease
HbA1c: Glycated hemoglobin
NHANES: National Health and Nutrition Examination Survey
NASH: Non-alcoholic steatohepatitis
HCC: Hepatocellular carcinoma
TE: Transient elastography
CAP: Controlled attenuation parameter
LSM: Liver stiffness measurement
NCHS: National Center for Health Statistics
HDL: High-density lipoprotein
HOMA-IR: Homeostasis model assessment of insulin resistance
BMI: Body mass index
WC: Waist circumference
ALT: Alanine aminotransferase
AST: Aspartate aminotransferase
ALP: Alkaline phosphatase
GGT: Gamma-glutamyl transpeptidase
TAGs: Triacylglycerols
